# Brachial Neuritis After a COVID-19 Booster Vaccination: A Case Report and Literature Review

**DOI:** 10.7759/cureus.45040

**Published:** 2023-09-11

**Authors:** Deeraj Loganathan, Nina Counsell, Vijay Kesavanarayanan, Ravi Badge

**Affiliations:** 1 Trauma and Orthopaedics, Warrington and Halton Hospitals NHS Foundation Trust, Warrington, GBR; 2 Orthopaedics and Trauma, University of Liverpool School of Medicine, Liverpool, GBR; 3 Radiology, Warrington and Halton Hospitals NHS Foundation Trust, Warrington, GBR

**Keywords:** steroid treatment neuropraxia, brachial neuropraxia, covid-19 vaccination complication, parsonage-turner syndrome, brachial neuritis

## Abstract

Idiopathic brachial neuritis also known as Parsonage-Turner syndrome is a rare neurological disorder characterised by pain and paraesthesia involving the shoulder girdle and upper limb, followed by weakness of the affected area. The cause is not very well understood and is often misdiagnosed leading to delayed treatment and long-term disability. There are many risk factors for the condition, with immunisations being accountable for as many as 15% of cases. In response to the coronavirus disease 2019 (COVID-19) pandemic, many companies have been producing and distributing COVID-19 vaccinations. To our knowledge, there have been 42 cases of brachial neuritis reported following COVID-19 vaccination to date. Here, we report a case of brachial neuritis following a patient’s fourth COVID-19 vaccination and present a review of the literature.

## Introduction

Parsonage-Turner syndrome (PTS) is a rare neurological disorder, with 1.6-3 cases identified per 100,000 persons per year [1]. It has an idiopathic cause, as described by Parsonage et al. in a series of case reports published in the Lancet in 1948 [2]. Also known as idiopathic brachial neuritis, acute brachial neuropathy, or neuralgic amyotrophy, PTS is characterized by a multitude of symptoms that can include pain, paresthesia, muscle weakness, and loss of reflexes of the shoulder girdle and upper limb. This likely results from an autoimmune reaction leading to inflammation of the brachial plexus. There is no obvious cause for the condition, however, medical literature does substantiate the theory that vaccination can be a trigger [3].

Many specific risk factors/triggers have been identified, including infection, immunisations, physiological stress, drugs, and direct trauma/iatrogenic complications, e.g., following orthopaedic procedures, coronary artery bypass surgery, and oral surgery [3]. A few theories propose that it is an immune-mediated response, which can be an abnormal reaction to a trigger which damages the brachial plexus in the process versus a possible infection directly involving the brachial plexus.

It can occur in healthy individuals with no co-morbidities, often starting as acute unilateral shoulder pain which can quickly amplify in severity and intensity. This can subsequently progress over days to weeks with progressive weakness, reflex changes, and sensory changes. These manifestations typically involve the shoulder girdle musculature and upper limb muscles [3].

PTS is a diagnosis of exclusion, given that it is determined via clinical findings [4], and it is oftentimes underdiagnosed. Several differential diagnoses that can present with similar symptoms should be considered, e.g., Pancoast tumours, thoracic outlet syndrome, cervical radiculopathy, entrapment neuropathies, mononeuritis multiplex, and direct brachial plexus trauma. The use of electromyography, nerve conduction studies, and magnetic resonance imaging (MRI) can aid in its detection.

## Case presentation

A 44-year-old male presented with a four-day history of progressively worsening atraumatic left-sided chest wall pain radiating to the left shoulder and arm with ipsilateral upper back involvement. The patient’s pain began insidiously. It was worse at night and did not respond to over-the-counter painkillers. The pain eventually became unbearable and emergency services were called in. The patient was seen by paramedics at home, who gave glyceryl trinitrate (GTN) spray for the chest-to-arm pain, which the patient reported to have temporarily eased the pain.

The patient described the chest pain as a crushing sensation and the arm pain being a pulsating shooting pain. The pain progressively worsened focally over the left scapula, radiating down the ulnar aspect of the forearm to involve the medial one and a half digits. This gradually spread over the dorsum of the forearm and the rest of the digits followed by paraesthesia. Any movement of the upper limb caused severe pain.

Blood tests for full blood count, C-reactive protein (CRP) and inflammatory markers, renal function, liver function tests, and troponin cardiac enzyme were all in the normal range.

Due to the chest pain migrating to the left side of the chest, the patient was admitted under the medical team to exclude any cardiac causes. An electrocardiogram (ECG), echocardiogram, computerised tomography (CT) angiogram, and aortic angiogram were performed with no abnormalities detected.

The patient was a right-hand dominant physician with a past medical history including hypertension and gastro-oesophageal reflux disease, for which he took losartan and omeprazole. The patient gave a history of coronavirus disease 2019 (COVID-19) vaccination to the left deltoid two weeks prior. This was the fourth vaccination dose, the first three being Pfizer vaccines and the fourth manufactured by Moderna. 

With cardiac and vascular conditions ruled out, a neurologist’s opinion was sought for possible neuralgic pain. Amitriptyline was commenced in addition to codeine, morphine, and ibuprofen with little effect. High-dose oral prednisolone of 40 mg daily was started while awaiting the findings of an MRI requested to assess for possible neuritis.

**Figure 1:**
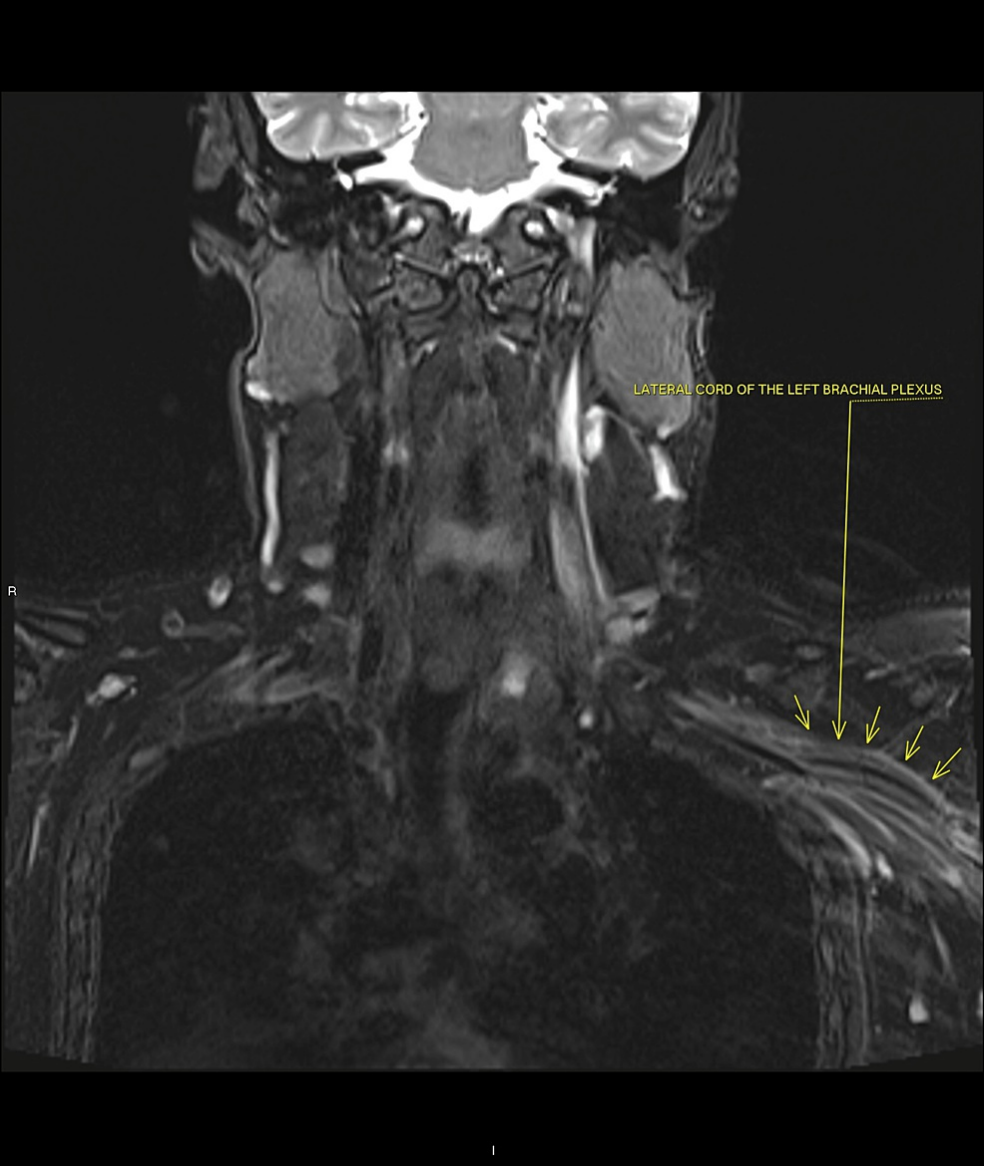
Thickening of lateral cord of brachial plexus indicating Parsonage-Turner syndrome.

**Figure 2:**
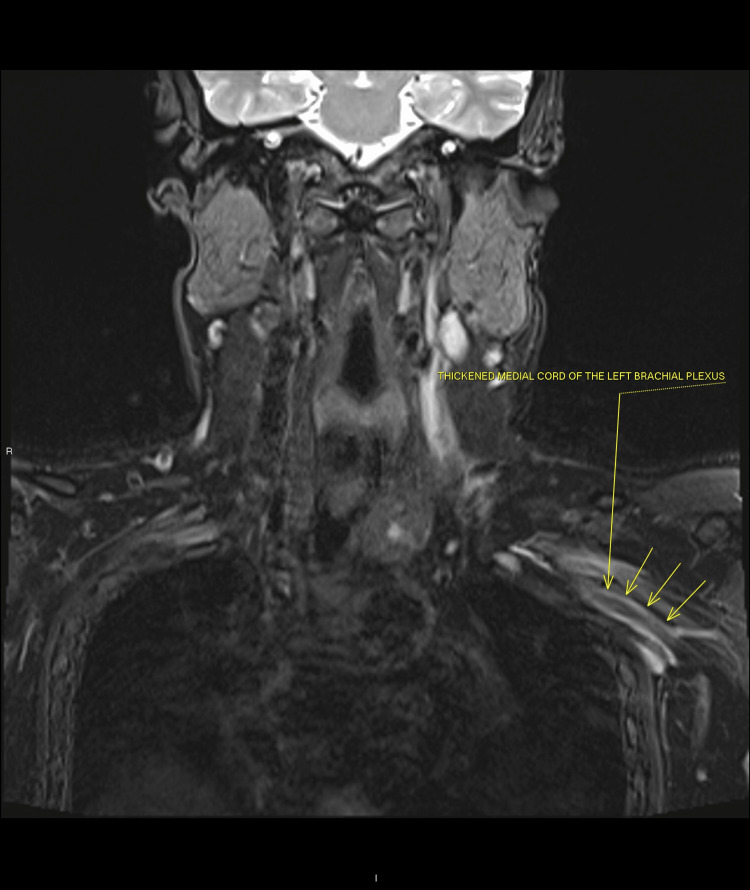
Thickening of the medial cord of brachial plexus indicating Parsonage-Turner syndrome.

The non-contrast MRI scan of the brachial plexus and cervical spine showed significant thickening and high signal change to the left brachial plexus including the cords and its branches (Figures 1, 2). The appearances were compatible with left brachial plexus neuritis/plexopathy. Nerve conduction studies were requested.

Electromyography studies (Table 1) and nerve conduction studies (Tables 2,3) six weeks from the onset of symptoms confirmed strong partial axonal left medial cord involvement of the brachial plexus. Left pronator teres showed active denervation changes along with unstable motor units. There was a slight asymmetry in the medial antebrachial cutaneous sensory response. There did not appear to be any involvement of the long thoracic nerve.

**Table 1:** Concentric needle electromyography results.

Concentric needle electromyography				
Muscle	Spontaneous	Motor units	Recruitment	Motor unit action potential (MUAP) amplitude mV
Left scalenus anterior	Silent	Long-duration polyphasic	Reduced	3
Left pectoralis major	Silent	Normal	Complete	1
Left flex dig superfic	Silent	Long-duration polyphasic	Reduced	1
Left abd pollicis brevis	Silent	Long-duration polyphasic	Reduced	3
Left inteross dors I	Silent	Normal	Complete	1
Left P teres muscle	Fibs +4	Unstable	Reduced	2
Left brachioradialis	Silent	Normal	Complete	1
Left ext dig communis	Silent	Normal	Complete	1
Left triceps	Silent	Normal	Complete	1
Left biceps	Silent	Normal	Complete	1
Left deltoideus post	Silent	Normal	Complete	1
Left trapezius	Silent	Normal	Complete	1
Left supraspinatus	Silent	Normal	Complete	1
Left infraspinatus	Silent	Normal	Complete	1

**Table 2:** Motor nerve conduction studies.

Nerve	Amp mV	Lat ms	CV m/s	F-wave min ms
Median motor left				
Wrist-APB	10.7	3.83		
Elbow-wrist	9.4	8.35	59.7	
Median motor right				
Wrist-APB	11.0	3.63		
Ulnar (FDI) motor left				
Wrist-FDI	10.2	4.38		
Bl. elbow-wrist	10.9	7.96	58.7	
Ab. elbow-Bl. elbow	11.6	10.2	44.6	
Ulnar motor left				
Wrist-ADM	9.9	2.63		
Bl. elbow-wrist	11.0	6.27	57.7	
Ab. elbow-Bl. elbow	11.1	8.31	49.0	
Ulnar motor right				
Wrist-ADM	11.3	2.38		
Bl. elbow-wrist	10.6	6.25	58.1	
Ab. elbow-Bl. elbow	10.3	7.83	63.3	

**Table 3:** Sensory nerve conduction studies.

Nerves	Onset lat ms	Peak lat ms	Amp uV	CV m/s
CTS program sensory left				
MII-wrist	2.63	3.22	15.7	51.3
MIII-wrist	2.83	3.33	20.7	49.1
UV-wrist	1.92	2.38	12.2	65.1
Med trans-wrist	1.77	2.29	30.0	45.2
Uln trans-wrist	1.34	1.71	16.9	59.7
CTS program sensory right				
MII-wrist	2.58	3.15	17.3	52.7
MIII-wrist	2.70	3.23	24.5	50.0
UV-wrist	2.00	2.36	11.7	60.5
Med trans-wrist	1.75	2.25	49.5	45.7
Uln trans-wrist	1.19	1.59	15.9	67.2
Cut antebr lat sensory left				
1-1	1.56	2.25	7.7	64.1
Cut antebr lat sensory right				
1-1	1.63	2.13	12.6	61.3
Cut antebr med sensory left				
1-1	1.48	1.86	3.2	67.6
Cut antebr med sensory right				
1-1	1.59	2.07	5.1	62.9
Radial sensory left				
Mid-forearm-snuff box	1.35	1.96	45.2	74.1

The patient was then reviewed by the neurologist. On examination, there was no wasting or overlying skin changes. Tenderness was noted along the shoulder blade and pectoralis major. Nil sensory deficit in any dermatomes was found. Weakness in the abduction of digits 2 and 4 with an absence of deep tendon reflexes in the left upper limb were positive findings noted.

The patient reported significant improvement in pain following the commencement of prednisolone, leading to the patient being able to wean off codeine and morphine. The neurologist discontinued amitriptyline and the patient was commenced on pregabalin 75 mg twice daily titrated to effect over the next two months. The prednisolone dosage was tapered down over two months from the initial dose of 40 mg. There was good resolution of symptoms and a return to baseline function at two months following the presentation.

## Discussion

Post-vaccination brachial neuritis is a recognised trigger of PTS in up to 15% of cases [1]. This phenomenon has been recognised following infections or vaccinations, particularly influenza, human papillomavirus, diphtheria, tetanus, and pertussis vaccines [3,5]. With the recent advent of the COVID-19 vaccine and its widespread administration, several cases of post-COVID-19 vaccination PTS are being reported. Overall, 69.7% of the global population has received at least one dose of a COVID-19 vaccine, which is over 13.3 billion doses recorded to date [6].

The pathophysiology behind this condition is not well understood. Given the nature of the causes, it is believed that genetic, environmental, and immune-mediated components contribute to its development [7]. Autoimmune reactions such as PTS can occur following mRNA vaccines such as the COVID-19 vaccines developed by Pfizer and Moderna. This is likely a form of molecular mimicry or bystander activation of immune-mediated components in response to the foreign substance. These vaccinations can elicit strong responses from type I interferons, which increases the potency of the vaccines; however, it can also lead to these autoimmune reactions [8].

Our literature search revealed that 42 cases of brachial neuritis have been published to date, with patients presenting following a COVID-19 vaccination.

In a review of the published cases, many of the vaccine-related cases did not follow the administration of an mRNA vaccine. The vaccines produced by AstraZeneca, Covishield, and Janssen in response to the coronavirus pandemic are adenoviral vector-based vaccines that use DNA material rather than mRNA. This group of vaccines has also shown to be a trigger for PTS; therefore, it is not necessarily from the type of vaccine used, but rather the subsequent immune-mediated response.

Of the 42 cases we found, including our own, it was noted that 64.3% of the patients were male and 37.5% were female. This aligns with the current belief that PTS affects more men than women on average [3]. The mean age across all patients was 52 years, ranging from 31 to 84 years of age. The most common presenting complaint was shoulder pain, accounting for 73% of the documented cases. 12 of the 42 cases did not mention the presenting symptoms. six cases presented with forearm/hand pain, weakness, and paraesthesia. Two cases also had chest wall pain which had no cardiac cause.

Overall, 50% of the cases were following a Pfizer/BioNTech vaccination, 29% following Astrazeneca, 19% of vaccinations were after Moderna, and 4.7% of cases were following Janssen. Although most cases reported were Pfizer/BioNTech, this is likely influenced by the proportion of vaccines delivered. Globally, 665 million doses of the Pfizer/BioNTech vaccine have been administered in comparison to 150 million for Moderna and 67 million for Oxford/AstraZeneca [6].

Further, 69% of the cases were attributed to an mRNA vaccination, and 31% followed an adenoviral vector-based vaccine. At the time of writing, over 92% of vaccines globally administered at the time were Pfizer/BioNtech and Moderna. Pfizer/BioNTech and Moderna are mRNA whereas Oxford/AstraZeneca are adenoviral vector-based vaccines. Given the disparity in the distribution of different vaccines, PTS cannot be definitively attributed to a specific type of vaccine used.

There appears to be no significance to the number of doses of the COVID-19 vaccine received and the development of PTS. We found that an equal number of cases developed PTS following their first or second doses (18 of 37 each). The remaining five cases did not document the number of previous doses. Our case had developed PTS following his first dose of the Moderna vaccination, with three previous Pfizer/BioNTech doses.

PTS was noted in 70.7% of the patients to be ipsilateral to the vaccination site. Five cases had contralateral-sided symptoms and two patients had bilateral PTS.

The onset of PTS symptoms has been recorded as early as 13 hours post-vaccination, with the longest onset being eight weeks after vaccination. Overall, 52% of the patients developed symptoms in the first week after the vaccination, with a further 23.8% in the second week.

Recovery from PTS takes a long time, often with conservative treatment and physical therapy being sufficient. A few studies have shown the administration of steroids for patients in the acute phase of PTS can shorten the duration of pain and time until recovery of paresis (28% within the first month compared to 6.3% in the untreated group; p = 0.001) [9].

Overall, 26 of the 42 cases reported were given steroids in comparison to 16 cases that were treated solely with analgesia and physiotherapy. In the cohort receiving steroids, it was documented that symptoms improved in 85% of the patients. In the remaining patients, documentation could not substantiate effectiveness. Most of these cases showed improvement in months one to four following the onset of symptoms. For the 16 patients who did not receive steroids, symptom improvement was noted to be between three and six months, with some taking longer.

From a review of the literature, it was difficult to assess the duration to resolution of all symptoms. Many patients were lost to follow-up, and many cases were published before full recovery. Overall, steroids did seem to help reduce the pain and accelerate the recovery period. The use of antiepileptic drugs, e.g., gabapentin, pregabalin, and amitriptyline, have also been shown to aid symptomatic relief.

## Conclusions

Brachial plexus neuritis is a rare and underdiagnosed condition which can be debilitating, with recovery lasting over two to three years. It is a recognised complication after infections or vaccinations. With the global vaccination response to COVID-19, this complication is increasingly being reported. There appears to be no definitive correlation to the type, brand, or number of doses of vaccination that can be concluded. Knowledge of COVID-19 vaccines as a risk factor may assist in the diagnosis and management of PTS. We recommend early investigation of patients with brachial plexus involvement and a recent history of vaccination. Brachial plexus MRI scans, nerve conduction studies, and EMG can help in the diagnosis. As there were no randomised controlled trials, anecdotal evidence suggests that corticosteroid use leads to a more rapid resolution of the painful phase of the illness, in particular, when used early in its course, although it does not seem to influence the final prognosis. Physiotherapy and the use of anti-epileptic drugs as neuropathic analgesia should also be considered for symptom relief.
